# Customizable optical tissue phantom platform for characterization of fluorescence imaging device sensitivity

**DOI:** 10.1117/1.JBO.28.8.086004

**Published:** 2023-08-30

**Authors:** Christopher Gibson, Arcturus Phoon, Ralph S. DaCosta

**Affiliations:** a University Health Network, Princess Margaret Cancer Centre, Toronto, Ontario, Canada; b University of Toronto, Department of Medical Biophysics, Toronto, Ontario, Canada

**Keywords:** imaging sensitivity, fluorescence imaging, phantoms, surgical guidance

## Abstract

**Significance:**

Optical tissue phantoms serve as inanimate and stable reference materials used to calibrate, characterize, standardize, and test biomedical imaging instruments. Although various types of solid tissue phantoms have been described in the literature, current phantom models are limited in that they do not have a depth feature that can be adjusted in real-time, they cannot be adapted to other applications, and their fabrication can be laborious and costly.

**Aim:**

Our goal was to develop an optical phantom that could assess the imaging performance of fluorescence imaging devices and be customizable for different applications.

**Approach:**

We developed a phantom with three distinct components, each of which can be customized.

**Results:**

We present a method for fabricating a solid optical tissue that contains (1) an adjustable depth capability using thin film phantoms, (2) a refillable chip loaded with fluorophores of the user’s choice in various desired quantities, and (3) phantom materials representative of different tissue types.

**Conclusions:**

This article describes the development of phantom models that are customizable, adaptable, and easy to design and fabricate.

## 1 Introduction

Optical tissue phantoms are necessary tools for the development of biomedical imaging applications, and they play a crucial role from proof-of-concept to clinical trials. These phantoms are used as reference materials to calibrate, optimize, characterize, standardize, and compare instruments. In addition, they are instrumental as inanimate and stable test materials to test and demonstrate new biomedical imaging principles before *in vivo* applications.

Different types of solid tissue phantoms have been described in the literature, such as polyester, polyurethane, silicone, gelatin, and agar phantoms.^1^^–^^9^ Although these reports describe effective phantom models, they do not explain whether or how they can be adapted to other applications. For example, tumor-to-normal contrast is critical in evaluating the imaging performance of a visible spectrum fluorescence (FL) imaging device. The appearance of the healthy tissue may be unique to a particular imaging device, thus necessitating a new phantom tailored to that device. In addition, these phantom models are limited in that they do not have a depth feature that can be adjusted in real-time. Finally, phantom tissue fabrication can be costly and laborious.^10^ Therefore, there is a need for optical phantoms that are easy to design and fabricate, provide an adjustable depth measurement capability, and contain fluorophores and tissue types of the user’s choice, making them more adaptable and customizable.

A significant challenge in developing FL imaging systems is complying with regulatory requirements regarding safety and efficacy. The following six critical features are desirable in an ideal FL-guided system: (i) real-time overlay of white light (WL) reflectance and FL images, (ii) FL-mode operation with ambient room lighting present, (iii) high sensitivity to fluorophore of interest, (iv) ability to quantify fluorophores *in situ*, (v) ability to image multiple fluorophores simultaneously, and (vi) maximized ergonomic use.^11^ Our goal was to develop and validate an optical phantom that could be used to assess the efficacy of FL imaging devices and their ability to meet the above recommendations. The results from these evaluations could potentially provide regulatory bodies with the required data to assess FL imaging technology and facilitate swift regulatory approval.

We present a method for fabricating a solid optical tissue phantom that, for the first time to our knowledge, contains (1) a real-time adjustable depth capability using thin film phantoms, (2) a refillable chip loaded with fluorophores of the user’s choice in various desired quantities, and (3) phantom materials representative of different tissue types. This article describes the development of a phantom platform that contains various amounts of absorbers, scatterers, and fluorochromes; is simply designed and fabricated to enable realistic evaluation of all types of FL imaging devices; and has the potential to inform a standard for FL imaging device characterization.

As an illustrative example, this article describes the development of phantoms to characterize an FL imaging device designed to image breast tissues under 405 nm irradiation following oral administration of 5-aminolevulinic acid (5-ALA). The device, called Eagle, includes 405 nm excitation light-emitting diodes (LEDs), a green (500 to 550 nm) and red (600 to 660 nm) imaging filter, an 8 MP image sensor, and a lens assembly with 2 to 15 cm autofocus. Eagle can be mounted on a stand with adjustable height for imaging *ex vivo* samples.

Modifications to the phantoms for different tissue types, devices, and excitation wavelengths are described in Sec. 2.4.

### 1.1 Phantom Requirements

Quantitative evaluation of a device’s conformance to the six expert-recommended FL imaging device features^11^ was the main driver of phantom development. In particular, designing the phantom to facilitate measurement of sensitivity to a fluorophore of interest—recommendation (iii)—was an essential requirement. The Eagle prototype device does not meet all six requirements, but the phantom was designed to evaluate such features. Section 4 describes how the phantom may be used to assess these features.

The phantom model consists of three customizable components: a tissue phantom base, a “chip” to hold the desired fluorophore(s) in various quantities, and thin tissue phantom films. The tissue phantom base is used to simulate the intrinsic optical properties and FL of tissues of a particular anatomy. The thin films are used to increase fluorophore depth below the imaging surface. This section discusses the requirements of each of the three phantom components to create a phantom model that can suitably test the performance of FL imaging devices.

#### 1.1.1 Tissue phantom material

**Fig. 1:**
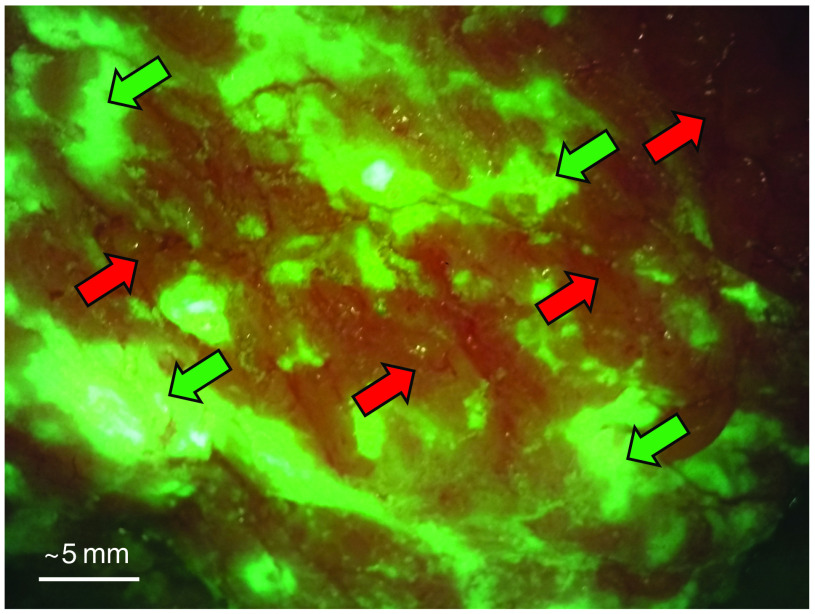
Breast surgical cavity imaged intraoperatively with the Eagle device from a distance of ∼3  cm. Based on past experience correlating breast FL images with histopathology^12^ and biopsy samples collected in this study, this FL image shows connective tissue in green (green southwest-facing arrows) and adipose tissue in orange-brown (red northeast-facing arrows).

The tissue phantom material (TPM) was designed to mimic the FL and optical properties of healthy breast tissues. FL images of breast tissues captured with the Eagle device in the clinical setting revealed that healthy fibrous connective tissues, composed mostly of collagen, appear green and adipose tissues appear orange-brown, as shown in Fig. 1. Therefore, separate connective and adipose TPMs (ATPMs) were required to best determine the impact of surrounding tissue types on tumor-to-normal contrast. Each model was designed to mimic the mean bulk optical properties—absorption and reduced scattering coefficients at 405 nm—and FL appearance of the respective tissue type to accurately represent the clinical tumor-to-normal contrast and light-tissue interactions.

##### Absorption

Connective TPM (CTPM): in the 300 to 600 nm range, the absorption coefficient of collagen increases exponentially with decreasing wavelength.^13^ This pattern was used to extrapolate the trend presented by Sekar et al.^14^ in the 500 to 800 nm range for collagen type I—the main component of connective tissue. An exponential fit with $R^{2}=0.9999$ revealed that $\mu_{a}=416.51\;{\mathrm{cm}}^{-1}e^{-0.014\lambda }$ for collagen between 300 and 600 nm. Therefore, the absorption coefficient of collagen under 405 nm excitation was calculated to be $1.44\;{\mathrm{cm}}^{-1}$.

ATPM: the wavelength dependence of the absorption coefficient for subcutaneous adipose tissue has been approximated to be $\mu_{a}(405\;\mathrm{nm})\approx 2.36\pm 0.25\;{\mathrm{cm}}^{-1}$ using the inverse adding-doubling method.^15^^,^^16^ We will assume that the absorption coefficient for breast adipose tissue at 405 nm also falls within this range.

##### Scattering

CTPM: The scattering of breast connective tissue (i.e., dense fibrous tissue) was estimated based on the optical properties of very dense breasts (i.e., breasts composed mainly of glandular and fibrous tissue as opposed to fat, n=37).^17^ Taroni et al.^17^ used the Mie theory approximation in Eq. (1),

$$\mu_{s}^{\prime }(\lambda )=a{\left(\frac{\lambda }{600\;\mathrm{nm}}\right)}^{-b},$$ (1)

to calculate the reduced scattering coefficient of breast tissues given tabulated values a and b. The error in the reduced scattering coefficient was calculated using Eq. (3):

$$\delta \mu_{s}^{\prime }(\lambda )=\sqrt{{\left[(\frac{\partial \mu_{s}^{\prime }}{\partial a})\delta a\right]}^{2}+{\left[(\frac{\partial \mu_{s}^{\prime }}{\partial b})\delta b\right]}^{2}},$$ (2)

$$={\left(\frac{\lambda }{600\;\mathrm{nm}}\right)}^{-b}\sqrt{{\left[\delta a\right]}^{2}+{\left[a\;\ln(\frac{\lambda }{600\;\mathrm{nm}})\delta b\right]}^{2}},$$ (3)

where δx is used to represent the error in variable x. In this case, δa and δb are provided by Taroni et al. as the standard deviation across measurements.^17^ The reduced scattering coefficient of highly dense breasts—and assumed to be the reduced scattering coefficient for breast connective tissues—was calculated to be $22.84\pm 4.28\;{\mathrm{cm}}^{-1}$ at 405 nm using a=15.12, b=1.05, δa=2.24, and δb=0.292.^17^

ATPM: Two equations, Eqs. (4) and (5), have been derived to characterize tissue $\mu_{s}^{\prime }$, which have been similarly effective for predictions in the spectral range of 400 to 1300 nm:^18^

$$\mu_{s}^{\prime }(\lambda )=a{\left(\frac{\lambda }{500\;\mathrm{nm}}\right)}^{-b},$$ (4)

and

$$\mu_{s}^{\prime }(\lambda )=a^{\prime }[f_{\text{Ray}}{\left(\frac{\lambda }{500\;\mathrm{nm}}\right)}^{-4}+(1-f_{\text{Ray}}){\left(\frac{\lambda }{500\;\mathrm{nm}}\right)}^{-b_{\text{Mie}}}].$$ (5)

Equation (4) scales the wavelength dependence of $\mu_{s}^{\prime }$ based on scattering behavior at a reference wavelength (500 nm), whereas Eq. (5) accounts for the individual contributions of the Rayleigh and Mie scattering. Mie scattering pre-dominates when particle size is comparable to or larger than the wavelength, which is not taken into consideration in Eq. (4).^18^ At 405 nm, the average scattering coefficient for adipose breast ranges from 11.8 to $15.6\;{\mathrm{cm}}^{-1}$, depending on the equation used (with a=10.6, b=0.520, $a^{\prime }=11.2$, $f_{\text{Ray}}=0.29$, and $b_{\text{Mie}}=0.089$).^19^ If we assume that most particles scattering light within adipose tissues are greater than 405 nm in diameter, this implies that ≥50% of the contribution to scattering comes from Eq. (5) and the remaining ≤50% from Eq. (4), then $13.6\;{\mathrm{cm}}^{-1}\leq \mu_{s}^{\prime }(405\;\mathrm{nm})\leq 15.6\;{\mathrm{cm}}^{-1}$.

##### Fluorescence

The appearance of the TPMs under FL imaging should closely match the appearance of the relative tissues as imaged in the clinic under the same imaging conditions to replicate the expected tumor-to-normal contrast. To assess the quality of the color matching, ΔE was used to measure the color difference between the phantom and images of breast tissues captured in the clinic. ΔE quantifies the perceptible difference between two colors, where ΔE≈1 is defined as the minimum perceptible color change by humans.^20^ ΔE was originally defined as the Euclidean distance between colors in the $L^{*}a^{*}b^{*}$ color space ($L^{*}$: lightness; $a^{*}$: red/green proportion, $a^{*}>0$ implies the color has more red than green; $b^{*}$: yellow/blue proportion, $b^{*}>0$ implies the color has more yellow than blue). Corrections were later made to the calculation to account for saturation, lightness, and relative weighting of color space components.^21^^,^^22^ This article uses the most up-to-date and accurate version of the ΔE calculation ($\Delta E_{2000}$, developed in the year 2000). ΔE was calculated using the inbuilt imcolordiff function in Matlab (MathWorks, Natick, Massachusetts, United States) with two colors as inputs.^21^^,^^22^ To calculate the contrast between two regions of interest (ROIs), the average colors of each ROI were calculated, and the ΔE between these two colors was subsequently calculated. For adequate color matching between the TPMs and breast tissues, the goal was to minimize ΔE as much as reasonably possible.

#### 1.1.2 Fluorophore chip

The fluorophore chip was designed to incorporate fluorophores, such as protoporphyrin IX (PpIX), indocyanine green, and quantum dots in various liquid quantities. The chip must therefore accommodate a fluorophore mixed with water, dimethyl sulfoxide (DMSO), blood, or other liquid solvents. The chip must be easy to fill with the fluorophore, washable, and refillable. The entire chip must be transparent to ensure that, when placed on top of the tissue phantom, the phantom is fully visible through the chip so as not to falsely influence the automatic gain control of the imaging device. The chip material itself must not be fluorescent.

The quantity of fluorophore present in each well is determined by the size of the well and the concentration of the fluorophore. Therefore, the chip must include wells for fluorophores in various concentrations and sizes. The concentrations chosen may be clinically relevant and the sizes determined by the clinical application and imaging device field of view (FOV), resolution, and imaging distance. For example, the Eagle device was designed to image FL from breast carcinoma at surgical margins. Therefore, the well size range may vary from the size of a few tumor cells (tens or hundreds of micrometers) to a few millimeters. To be detectable, the smallest wells should be larger than or equal to the imaging device’s resolution at the nearest imaging distance. Wells of a given size must all be equidistant from the center of the chip, which should also be the center of the imaging device’s FOV. The FL of a well should not affect the FL emitted from an adjacent well.

The chip thickness may vary based on the desired application. For example, when simulating FL from a solid tumor, the depth of the “tumor” may be some centimeters thick. Instead, when mimicking a clinical scenario whereby a small area of residual carcinoma is present at the imaged surface, the depth of the chip may only need to be a few millimeters.

The wells need to be covered to prevent evaporation and accidental spillage. The well covering should be transparent and non-fluorescent under 405 nm excitation.

#### 1.1.3 Thin films

Thin films must be placed over the fluorophore chip to simulate depth below the surface, e.g., to mimic the effect of invasive carcinoma situated directly below healthy tissue. Therefore, the films are subject to the same optical and FL requirements of the TPMs, and separate adipose and connective versions must be used. The thickness should be sufficient to deepen the fluorophore in the chip by various depths up to the maximum theoretical penetration depth of the relevant light wavelengths in the tissue type of choice. The films must have sufficient mechanical strength to be removed from their storage container and placed on the fluorophore chip without tearing or deforming. The films need to sit flat when placed on the chip and fully cover the wells of the chip.

### 1.2 Materials and Fabrication

#### 1.2.1 Tissue phantom material

The TPM was based on the material developed by Pleijhuis et al.^5^ This phantom consists of a tris buffer and a gelatin mixture. The tris buffer helps to maintain the pH and stability of the phantom. The sodium azide additive in the buffer assists in preserving the model by acting as an antibacterial agent. To replicate optical properties, the gelatin mixture includes intralipid (IL) and hemoglobin (Hb). Additional materials and dyes may be used to influence the optical properties and FL appearance. The gelatin allows the phantom mixture to set into a long-lasting, reproducible shape. These materials are mixed in liquid form and solidify while cooling.

Previous studies have used gelatin-based phantoms and adjusted the optical properties with varying Hb and IL concentrations.^5^^,^^23^ Hb concentrations can be used to adjust for the absorption of incoming photons from a light source, and IL (a fat emulsion) can be used to adjust for scattering.^5^^,^^24^ The protocol outlined by Pleijhuis et al. detailed the fabrication of a tissue-like breast phantom to test a near-infrared FL imaging modality with excitation wavelength between 750 and 800 nm.^5^ Accordingly, the quantities of absorbers and scatterers were adjusted to mimic the optical properties of each breast tissue type at 405 nm. A gelatin concentration of 8% ($80\;\frac{\mathrm{mg}}{\mathrm{mL}}$) was used in these TPMs. Gelatin concentrations as low as 4% were tested but were found to be considerably more delicate and malleable when set. 8% gelatin was found to provide sufficient mechanical strength with a modest material cost.

To fabricate the TPM, gelatin (Sigma-Aldrich, Darmstadt, Germany) suspended in tris buffer^5^ was heated on a hot plate to 50°C then cooled to 35°C under constant stirring. Appropriate quantities of absorbers and scatterers were then stirred into the phantom. Reverse osmosis (RO) water was added to dilute the reagents to their final concentrations.

The concentration of IL (Sigma-Aldrich) added to the phantom affects the scattering properties of the tissue phantom material. Michels et al. calculated the wavelength-dependent reduced scattering coefficient to be

$$\mu_{s}^{\prime }(\lambda )=y_{0}+a\lambda +b\lambda^{2},$$ (6)

where $y_{0}$, a, and b are coefficients tabulated for 10%, 20%, and 30% IL.^25^ Vardaki et al. used this data to calculate the concentration of IL required for their application at 830 nm.^23^ Their method was replicated to calculate the IL concentration at 405 nm for the CTPM and ATPM.

The concentration of absorbers in the phantom was chosen to match the absorption properties of the relevant breast tissue at 405 nm. For the CTPM, Hb (BioShop, Burlington, Ontario, Canada) was used as the absorber. The saturation of breast tissue is reported to be between 60% and 80%.^18^ 70% saturated Hb was therefore used throughout calculations. The concentration of Hb was calculated using the following equation:^26^

$$C=\frac{64,500\;\frac{g}{\mathrm{mol}}\mu_{a}(\lambda )}{\ln(10)\varepsilon (\lambda )},$$ (7)

where $\mu_{a}(\lambda )$ is the absorption coefficient in ${\mathrm{cm}}^{-1}$ at wavelength λ, $\varepsilon (\lambda )=0.7\varepsilon_{{\mathrm{HbO}}_{2}}(\lambda )+0.3\varepsilon_{\mathrm{Hb}}(\lambda )$ is the extinction coefficient of 70% saturated Hb in $\frac{{\mathrm{cm}}^{-1}}{M}$, C is the concentration of Hb in $\frac{g}{L}$, and $64,500\;\frac{g}{\mathrm{mol}}$ is the molecular weight of Hb. The extinction coefficient ε(λ) was calculated to be $\varepsilon (405\;\mathrm{nm})=310,610.8\;\frac{{\mathrm{cm}}^{-1}}{M}$ using tabulated $\varepsilon_{{\mathrm{HbO}}_{2}}(\lambda )$ and $\varepsilon_{\mathrm{Hb}}(\lambda )$ for oxygenated and deoxygenated Hb, respectively.^26^

The gelatin and IL in the phantoms cause the CTPM to appear green, as desired, when imaged with the Eagle FL device. These materials are also required in the ATPM to enable solidification and mimic scattering. Therefore, changing the color of the ATPM to match the orange-brown adipose tissues as seen in the clinic (Fig. 1) required the addition of new materials. Direct red 81 (DR81), a red azo dye that preferentially absorbs green wavelengths within the visible spectrum,^27^ and yellow food coloring (YFC)^28^ were added as absorbers to the ATPM. Concentrations of absorbers Hb, DR81 (Sigma-Aldrich), and YFC (McCormick Canada, London, Ontario, Canada) in the ATPM were determined by measuring the full-spectrum transmission %T(λ) of each absorber at three initial concentrations in triplicate with a Varian Cary 300 spectrophotometer (Agilent Technologies, Santa Clara, California, United States) using a d=1  cm path length cuvette. The average of the triplicate transmission was used to calculate $\mu_{a}(\lambda )$ for each absorber at the tested concentration using the following equation:

$$\mu_{a}(\lambda )=-\frac{1}{d}\;\ln(\frac{\%T(\lambda )}{100\%}).$$ (8)

The error in $\mu_{a}(\lambda )$ was calculated using the following equation:

$$\delta \mu_{a}(\lambda )=|\frac{\partial \mu_{a}}{\partial (\%T)}|\delta (\%T)=\frac{100}{d\%T}\sigma ,$$ (9)

where δ(%T)=σ is the standard deviation of the triplicate %T measurements. The relationship between $\mu_{a}(405\;\mathrm{nm})$ and concentration was estimated for each absorber, assuming linear trends that cross through the origin. The ratio of absorbers was determined iteratively by fabricating phantoms, imaging them, measuring ΔE relative to human breast adipose tissues, and modifying the absorber concentrations to decrease ΔE. The ratio of absorbers r used to match the color of adipose tissues was used to estimate the absorber concentrations $C_{i}$ required to meet the $\mu_{a}(405\;\mathrm{nm})$ target using Eq. (12):

$$\mu_{a}(405\;\mathrm{nm})=\sum_{i}C_{i}\mu_{ai}(405\;\mathrm{nm}),$$ (10)

$$\mu_{a}(405\;\mathrm{nm})=q\sum_{i}r_{i}\mu_{ai}(405\;\mathrm{nm}),$$ (11)

$$C_{i}=\frac{\mu_{a}(405\;\mathrm{nm})}{\underset{i}{\sum }r_{i}\mu_{ai}(405\;\mathrm{nm})}r_{i},$$ (12)

where i=Hb, DR81, YFC indicates the absorber, $C_{i}$ is the concentration of absorber i, $\mu_{ai}$ is the estimated absorption coefficient of absorber i per unit concentration, $r_{i}$ is the relative proportion of absorber i, and q is the base quantity relative to which all $C_{i}=r_{i}q$ are calculated. Then, the $C_{i}$ were used to fabricate a modified prototype phantom: since $\mu_{s}^{\prime }\gg \mu_{a}$, an ATPM with the IL substituted by RO water was used to eliminate scattering. The transmission of the modified phantom was measured and q was adjusted using the prototype phantom’s absorption coefficient to conform to the $\mu_{a}(405\;\mathrm{nm})$ requirement. The adjusted base quantity q was used to recalculate each $C_{i}$, and a new phantom was made using these concentrations. This phantom’s absorption coefficient and color were measured as a final verification of conformance to the $\mu_{a}(405\;\mathrm{nm})$ and ΔE requirements.

#### 1.2.2 Fluorophore chip

Polydimethylsiloxane (PDMS) was selected as the chip material due to its low FL.^29^ The PDMS was purchased in a kit (SYLGARD™ 184 Silicone Elastomer Kit, Dow Chemical, Midland, Michigan, United States) that included a base and curing agent. The PDMS was set to cure in a 3D-printed mold to form the shape and size of the chip. The mold was printed using a stereolithography thermoset (Accura^®^ Xtreme™, 3D Systems, Rock Hill, South Carolina, United States). Holes, sized to create wells of diameters ≲1  mm, were milled and reamed into the printed mold base to a depth of 4 mm to form interference fits with needle segments. Needles (BD, Franklin Lakes, New Jersey, United States) were clamped and cut into ∼1.5  cm-long segments using a Dremel tool. Pointed ends were ground and smoothed with a metal file to a ∼1  cm length before insertion into the mold base. PDMS was prepared by combining the base and curing agent in a 10:1 ratio. This mixture was stirred for 5 min and degassed in a vacuum desiccator for at least 20 min. 3.7 g of the PDMS mixture was poured into the mold and degassed for at least 10 min. The PDMS in the mold was set in a fume hood for 72 to 96 h until cured. Once cured, the largest wells of the chip were painted, using a small pointed cotton swab, with an opaque black colorant to minimize light leakage from these wells into neighboring wells. [For compatibility with water-soluble fluorophores, nail polish was used; for DMSO-soluble fluorophores (e.g., PpIX), oil-based paint was used. It may also be possible to use oil-soluble fluorophores with water-based paint, but this has not been tested.]

The chip uses capillary tubes to create the desired inner diameters (1Ds) of the wells. Fused silica capillary tube (Molex, Lisle, Illinois, United States and AAdvance Instruments, Stony Brook, New York, United States) segments equal in length to the thickness of the chip were inserted halfway into the holes created in the chip by the needles designated for the tubes. The tubes were cleaved using a cleaving stone (Molex). The halfway-protruding tubes were then able to be filled by lowering the chip onto a reservoir such that the protruding tubes were dipped into the channels of the reservoir, which contained desired fluorophore concentrations.

After filling the chip with the fluorophore and pressing the tubes in to create a flush surface, one circular face of a fused silica wafer (Custom Glass and Optics, Williamsburg, Virginia, United States) was coated with a thin layer of silicone grease (Moen, North Olmsted, Ohio, United States). The PDMS chip was then placed on the greased face of the wafer with light pressure applied to the PDMS to squeeze out air bubbles that were trapped between the PDMS and the wafer. The grease created a watertight seal between the PDMS and fused silica wafer.

#### 1.2.3 Thin film phantoms

To make the thin films, aluminum slabs (McMaster-Carr, Elmhurst, Illinois, United States, dimensions 15  cm×15  cm×0.6  cm, mass: 396±2  g) were moistened with a wet paper towel. A 10  cm×15  cm sheet of Parafilm (Bemis, Neenah, Wisconsin, United States) was flattened against the dampened face of each of the slabs to create a flat surface against which the phantom could set. Two ∼17  cm-long feeler gauge (McMaster-Carr) segments of equal thickness were placed on the Parafilm near opposite edges of one slab. The feeler gauge thicknesses used were between 200  μm and 1 mm in 100  μm increments, resulting in films created at each of these thicknesses.

Next, liquid TPM was pipetted into the center of the Parafilm. The amount of TPM increased with the thickness t of feeler gauge used: $3\;\mathrm{mL}+\frac{1.5\;\mathrm{mL}}{100\;\mu m}(t-200\;\mu m)$, up to a maximum of 7 mL. The top aluminum slab was then applied to compress the TPM to the feeler gauge thickness. The entire mold was cooled to ∼4°C to allow the phantom to set. Once set, the aluminum slabs and one sheet of Parafilm were removed. If holes in the film were observed, they were filled with liquid TPM. The Parafilm was then reapplied, flattened, and the phantom stored in the dark at 4°C until set.

Next, the films were mounted on a fused silica wafer by placing the wafer on the film, cutting the film around the wafer with a scalpel, and lifting the film and wafer off the Parafilm. Edges of the film were trimmed to match the diameter of the wafer. The wafer and film were wrapped in a sheet of Parafilm to prevent the film from drying out before use. This was repeated for all desired thicknesses, and the films mounted on wafers and wrapped in Parafilm were stored in an airtight container in the dark at 4°C until used.

## 2 Results

### 2.1 Phantom Material and Optical Properties

#### 2.1.1 Absorption

##### Connective tissue phantom model

**Fig. 2:**
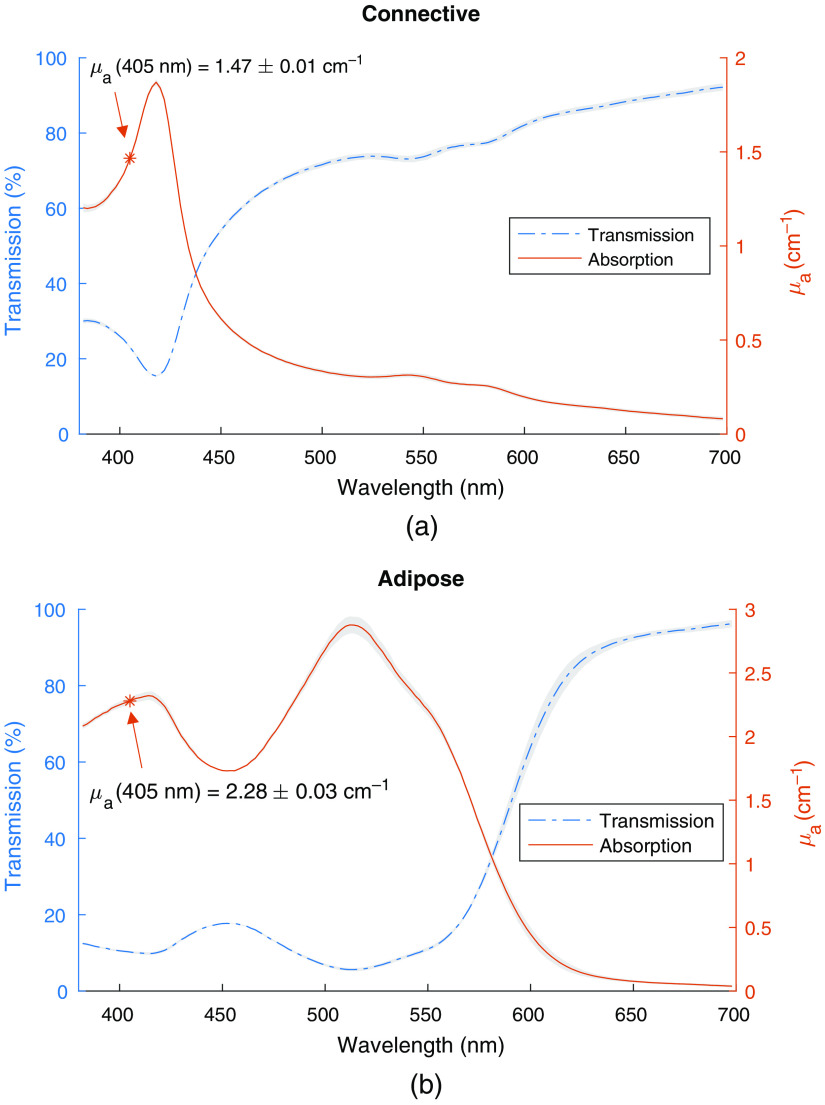
Measured %T and calculated $\mu_{a}$ for the absorbing materials in the (a) CTPM and (b) ATPM. The gray bands around the transmission spectrum represent the standard deviation from the triplicate transmission measurements, with error propagated through to the absorption coefficient using Eq. (9).

Solving Eq. (7) using $\mu_{a}=1.44\;{\mathrm{cm}}^{-1}$ produces a Hb concentration of $C=0.130\;\frac{g}{L}$ (0.0130%). This concentration was adjusted to $0.135\;\frac{g}{L}$ (0.0135%) following empirical measurement of the absorption coefficient of the CTPM, resulting in $\mu_{a}(405\;\mathrm{nm})=1.47\pm 0.01\;{\mathrm{cm}}^{-1}$. The transmission and absorption coefficient spectra are plotted in Fig. 2(a). The absorption coefficient was calculated using Eq. (8), and the error, represented by the grey bands around the absorption coefficient spectrum, using Eq. (9).

##### Adipose tissue phantom model

To meet the requirements for absorption coefficient and appearance under FL imaging (Sec. 2.1.3), the ATPM uses a combination of Hb, DR81, and YFC for absorption. Iterating to find a combination of absorbers that satisfied both the absorption and FL requirements resulted in $[\mathrm{Hb}]=0.041\;\frac{g}{L}$ (0.0041%), [YFC]=0.030%, and $[\mathrm{DR}81]=0.033\;78\frac{g}{L}$ (0.003378%) comprising the absorbers in the ATPM. Combining these reagents, we found that $\mu_{a}(405\;\mathrm{nm})=2.28\pm 0.03\;{\mathrm{cm}}^{-1}$ [see Fig. 2(b) for the full spectrum], which is within the target range.

#### 2.1.2 Scattering

##### Connective tissue phantom model

By replicating the method presented by Vardaki et al. to calculate IL concentration corresponding to a reduced scattering coefficient^23^ at 405 nm, we found that $\mu_{s}^{\prime }=1.9035\;\frac{{\mathrm{mm}}^{-1}}{\%}C$ (linear fit with $R^{2}=0.9805$). For $\mu_{s}^{\prime }=22.84\;{\mathrm{cm}}^{-1}$, the corresponding IL concentration is C=1.20% at 405 nm. However, mixing IL with gelatin can effectively decrease $\mu_{s}^{\prime }$ from IL by a factor of approximately $\frac{80}{220}$ at 8% gelatin.^30^ Given that $\mu_{s}^{\prime }$ scales linearly with IL concentration, the actual IL concentration is 3.30%. Since the only contribution to scattering in the phantom is from IL, and the relationship between reduced scattering coefficient and IL concentration is well-documented,^23^^,^^30^ the scattering coefficient was not empirically measured.

##### Adipose tissue phantom model

Following a similar method to the CTPM, the IL concentration was determined to be 2.1% in the ATPM for $\mu_{s}^{\prime }(405\;\mathrm{nm})=14.53\;{\mathrm{cm}}^{-1}$.

#### 2.1.3 Fluorescence

##### Connective tissue phantom model

**Fig. 3:**
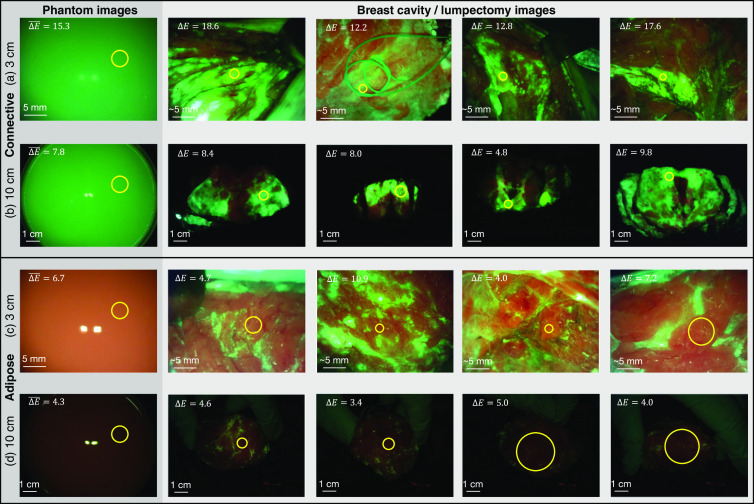
TPMs imaged in comparison to human breast tissues. Images of the TPMs are shown on the left and four images from the clinic—captured of the same tissue type from the same imaging distance—on the right. Images from the clinic were captured from 10 cm of lumpectomy samples and from ∼3  cm of surgical cavities. (a) CTPM from 3 cm. The mean ΔE between the average colors within the yellow circle in this image and the yellow circles in the clinic images are noted in the top left. Reflections from the Eagle device’s two 405 nm LEDs are slightly visible near the center of the image. To the right are four clinical reference images from ∼3  cm used for ΔE calculation. The ROIs used for ΔE calculation are shown in yellow circles. ΔE values shown here are relative to the CTPM. (b) CTPM from 10 cm. (c) ATPM from 3 cm. (d) ATPM from 10 cm.

**Fig. 4:**
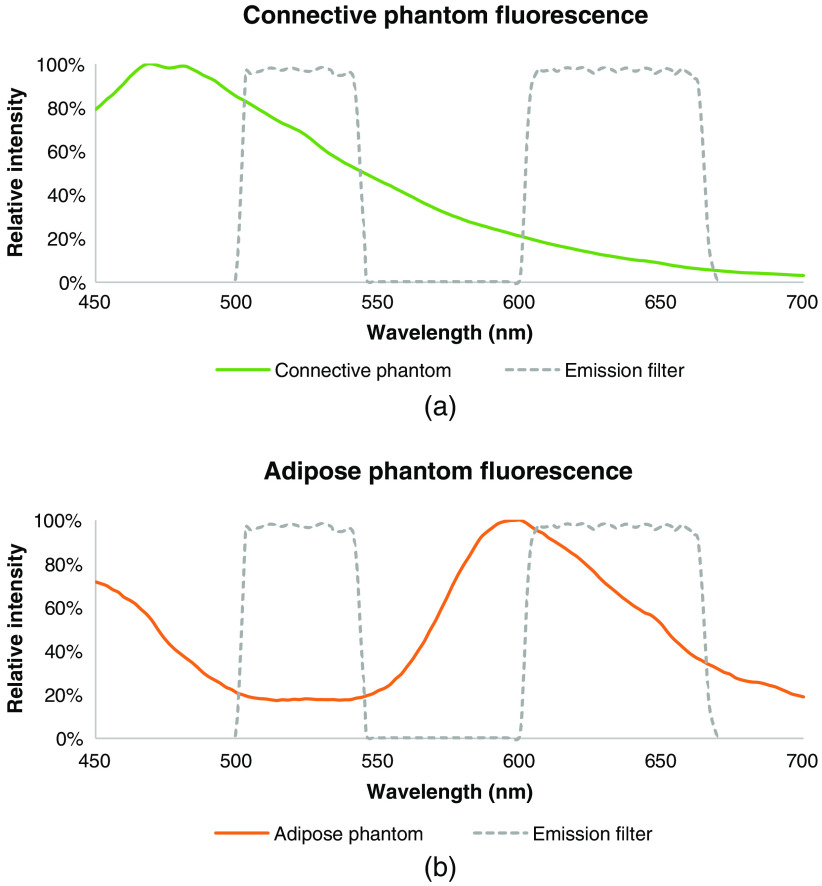
FL emission spectrum from the (a) CTPM and (b) ATPM when excited at 405 nm, plotted with the Eagle device’s emission filter. The absorption of green light (500 to 550 nm) by DR81 in the ATPM is evident.

The materials chosen to mimic the absorption and reduced scattering coefficients of the CTPM exhibit relatively similar FL to that of the connective tissues found in the breast. The accuracy of the color matching was evaluated by capturing images of the CTPM from 3 and 10 cm and comparing the color to that of breast connective tissues imaged during a clinical trial (ClinicalTrials.gov Identifier: NCT01837225) from approximately these distances. The images of the CTPM, captured from 3 and 10 cm, respectively, are shown in the left columns of Figs. 3(a) and 3(b). A WL image of the CTPM is shown in Fig. 6(a). ΔE was calculated as a measure of the color difference between the phantom and a region of connective tissue in the corresponding clinical image captured from the same distance. This was repeated for four clinical reference images of the breast cavity at 3 cm [Fig. 3(a)] and lumpectomy at 10 cm [Fig. 3(b)]. Color contrast was calculated between the mean color within the circles in the phantom images and each of the four corresponding clinical images. The mean color contrast, $\bar{\Delta E}$, between the CTPM and breast connective tissues is 15.3 at 3 cm and 7.8 at 10 cm. The FL emission spectrum for the CTPM, when excited by 405 nm light, is shown in Fig. 4(a). This spectrum was collected with a Photon Technology International fluorometer, and the FL of a blank cuvette was subtracted.

##### Adipose tissue phantom model

The ATPM is shown imaged with the Eagle device from 3 and 10 cm in Figs. 3(c) and 3(d), respectively. A WL image is shown in Fig. 6(b). The mean color contrast, $\bar{\Delta E}$, between the ATPM and breast adipose tissues is 6.7 at 3 cm and 4.3 at 10 cm. The FL emission spectrum for the ATPM, when excited by 405 nm light, is shown in Fig. 4(b).

### 2.2 Fluorophore Chip Design and Use

**Fig. 5:**
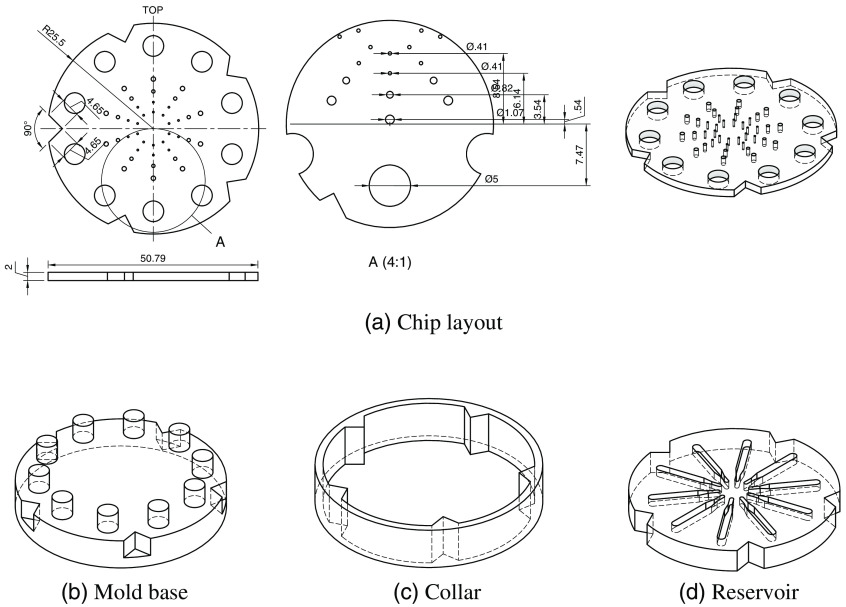
(a) Layout of wells on the fluorophore chip as molded. Dimensions in mm. Designs of the (b) mold base, (c) collar, and (d) reservoir for 3D printing.

**Fig. 6:**
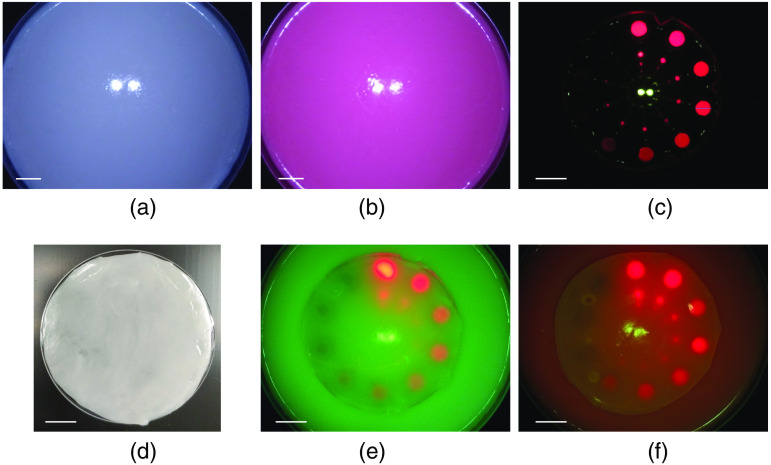
Images of the fabricated phantom. WL images of the (a) CTPM and (b) ATPM from 10 cm. (c) The fluorophore chip filled with 630 nm quantum dots. The concentrations are the same in each radial column of the chip. This FL image was captured from 10 cm. (d) Top view of a connective tissue 0.9-mm-thin film mounted on a 5.5-cm-diameter fused silica wafer. Examples of assembled (e) CTPM and (f) ATPM phantoms imaged from 10 cm with 400-μm- and 800-μm-thick films placed on the CTPM and ATPM, respectively. Scale bars are 1 cm.

The fluorophore chip was designed to hold fifty quantities of a fluorophore with five radial arrays of ten wells each with diameters 100  μm, 250  μm, 500  μm, 1 mm, and 5 mm, as shown in Fig. 5(a). The chip is 2 mm thick. An example of a filled chip is shown in Fig. 6(c).

The chip is made of PDMS, which is virtually non-FL. The custom mold used to shape the PDMS has two main parts: the mold base and the collar. The protruding cylinders in the mold base, shown in Fig. 5(b), create the 5 mm wells. As described previously, needles were inserted into holes drilled into the mold base to create the smaller wells. In total, 40 holes were drilled in the mold for needles, which formed stalks in the mold to create the four innermost rings of wells in the PDMS. The holes were drilled to achieve interference fits with 19G (1.067 mm outer diameter), 21G (0.819 mm), and 27G (0.413 mm) needle sizes. Capillary tubes create the required IDs of the three inner rings of the chip (IDs 100, 250, and 500  μm for the three innermost rings, with outer tube diameters 375, 355, and 750  μm, respectively). The tubes were cleaved in ∼2  mm segments to match the thickness of the chip using a cleaving stone.

The collar, shown in Fig. 5(c), molds the sides of the chip. It is removable to simplify the lifting process of the PDMS from the mold once set. The mold base and collar were 3D printed in high resolution with a smooth surface finish.

#### 2.2.1 Cleaning and reusing

The chip can be reused by rinsing out the fluorophore and cleaning off residual grease from the wafer. To thoroughly clean the chip and prepare it for subsequent uses, it can be rinsed and soaked in RO water for ≥12  h. The wafers can also be scrubbed with hexanes or unscented liquid hand soap and a non-abrasive sponge or wipe to remove grease. Compressed air blown through the capillary tubes can remove any liquid. Placing the chip in a vacuum desiccator for 2 h will further ensure the removal of bubbles and any moisture trapped in the capillary tubes. A light microscope can be used to verify that no tubes are clogged.

### 2.3 Thin Films

The thin films were developed in thicknesses from 200  μm to 1 mm in 100  μm increments. Due to the thickness of the films, mounting them on fused silica wafers (50.8 mm diameter and 0.7 mm thickness) in advance simplified swapping the films while imaging. An image of the top of a connective tissue film is shown in Fig. 6(d).

### 2.4 Phantom Variations

#### 2.4.1 Tissue phantom variations

Due to the nature of the phantom fabrication, several variations can be made to the TPMs for other applications. The absorbing and scattering materials can be adjusted to mimic other tissue types at different wavelengths by developing new requirements as done in Sec. 1.1.

##### Addition of dyes

The TPMs include RO water to balance the concentrations of the gelatin, absorbers, and scatterers. The water can be replaced with water-soluble dyes, fluorophores, absorbers, scatterers, or other solutions to introduce additional properties to the phantom. Alternatively, the phantom can be soaked in a water-soluble dye, such as methylene blue (MB), to simulate the effect of the dye being injected subcutaneously during surgery and absorbed by the breast tissue, for example. Such data may be used to predict and characterize the impact of previously untested clinical scenarios.

##### Composite tissue phantom

**Fig. 7:**
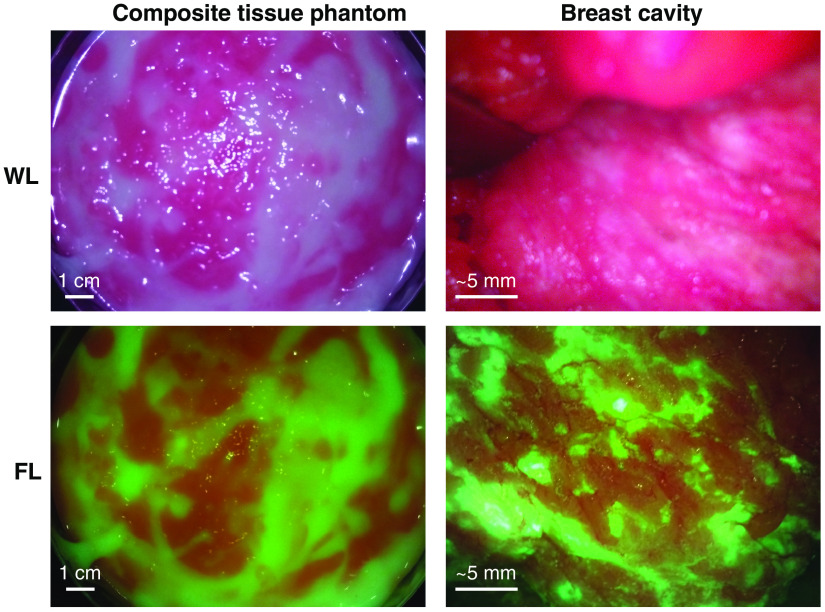
Composite breast tissue phantom made by combining cooled CTPM and ATPM materials. Images were captured under WL and FL in the top and bottom rows, respectively. Images of the composite tissue phantom, shown on the left, were captured from a 10 cm imaging distance. On the right, WL and FL images of a breast surgical cavity are shown for comparison.

A composite tissue phantom can be fabricated by combining two or more separate TPMs as they are cooling. For example, a composite breast tissue phantom can be fabricated by pouring ATPM and CTPMs into a desired mold in layers, which are a few millimeters thick. After a layer is partially cooled (∼5  min at 4°C and 35% humidity), more TPMs may be poured into the mold, forming another layer. The configuration and quantities of the different TPM may be chosen ad hoc to reflect the desired application. Typically, the ATPM and CTPM are fabricated by heating the gelatin to 50°C, allowing to cool to 35°C, and mixing with absorbers and scatterers. Cooling the mixture beyond 35°C, to between approximately 28°C and 32°C, before pouring into a mold will increase the viscosity of the phantom, permitting greater control over the pattern of tissue materials. Allowing the TPMs to further cool to between approximately 24°C and 28°C before pouring may result in some solidification and clumping of the gelatin. This can be used to create a textured surface by wiping away unsolidified TPM in the top layer and subsequently allowing it to fully cool to 4°C. The result is a phantom with a mottled connective and adipose tissue appearance, as shown in Fig. 7, with or without a textured surface as desired. Alternatively, phantoms with discrete heterogeneous layers (e.g., including skin) can be produced by sequentially pouring layers into a mold and allowing them to set before adding the next layer. Solid inclusions can be introduced by allowing material to set around the inclusions. Such phantoms may be useful for ad hoc device testing where the heterogeneous makeup of tissue is valuable, such as when comparing alternative device components or features.

##### Form factors

Finally, molds can form the tissue phantom into different form factors, which are useful when the anatomic geometry is important (e.g., for training purposes).

#### 2.4.2 Fluorophore chip variations

In addition to the TPMs, the fluorophore chip can be modified for other applications.

##### Addition of dyes

Dyes can be incorporated into the PDMS to simulate absorption of the fluorophore emission by dyes in adjacent tissues, which were easily accomplished using a non-polar dye, since PDMS is non-polar and “like-dissolves-like.” However, the complexity increases with a polar dye, such as MB, which requires the use of an intermediary solvent or micelles to facilitate dissolution of the dye into the PDMS. For example, we found that MB could be mixed into PDMS by first dissolving MB in glycerol and then mixing the MB/glycerol solution into PDMS.

##### Chip fabrication

The PDMS chip can be fabricated in various thicknesses by adding corresponding volumes of PDMS to the mold. The wells’ sizes, quantities, and locations can also be varied by modifying the mold and needle sizes.

### 2.5 Sample Imaging Device Characterization Procedure

The phantom contains three parts: a tissue phantom base, a fluorophore chip, and thin films. This section outlines the use of these phantom components to characterize the imaging performance of an imaging device, with the Eagle device used as an example.

#### 2.5.1 Phantom assembly

The tissue phantom bases were fabricated as described in Sec. 1.2. 50 mL of each of liquid connective and ATPMs were poured into 10 cm-diameter Petri dishes, covered with the Petri dish lids, and allowed to set in the dark at 4°C and 35% humidity for 24 h. Connective and adipose thin films in thicknesses between 200  μm and 1 mm in steps of 100  μm were fabricated, mounted on fused silica wafers, wrapped in Parafilm, and stored according to Sec. 1.2.

##### Fluorophore chip filling

The Eagle device was designed to image PpIX in breast carcinoma cells. 630 nm quantum dots were used for this demonstration instead of PpIX to avoid photobleaching. The fluorophore chip, collar, and reservoir were fabricated as described in Sec. 1.2. To fill the chip, 55  μL each of selected quantum dot concentrations (450, 300, 180, 120, 80, 55, 40, 25, 16, and $0\;\frac{\mu g}{\mathrm{mL}}$) were loaded into channels of the reservoir with concentrations decreasing in the clockwise direction. The collar was then placed on the chip, and the chip was placed over the reservoir, allowing the collar to align the chip’s protruding capillary tubes with the channels of the reservoir. The location of the control well was marked with a waterproof marker on the chip. Silicone grease was applied to one side of a fused silica wafer. The chip was flipped over an empty reservoir with the tubes pointing upward, again allowing the collar to align the tubes with the reservoir channels. The tubes were pushed into the chip using precision forceps to create a flush surface. The collar was removed, and the flattest side of the chip was placed on the greased face of the fused silica wafer. Precision forceps were used to gently press down on the chip to remove any air bubbles. 2.5 and 50  μL of each quantum dot concentration were loaded into the 1 mm- and 5 mm-diameter wells, respectively, with concentrations increasing in the counter-clockwise direction.

#### 2.5.2 Imaging

To image the phantom, the Petri dish containing the adipose tissue phantom base was taped to the center of a black polyoxymethylene sheet (25  cm×30  cm) and the filled fluorophore chip was placed on the phantom base. The black sheet was placed on the optical board of the Eagle imaging stand and the Eagle device was placed on the stand. Another fused silica wafer was placed on top of the chip and the vertical distance between the top of the wafer and the distal tip of the imaging distance was adjusted to 10 cm. The black sheet was then shifted to center the phantom in the device’s FOV and taped to the optical board to prevent movement of the phantom between images.

An FL image was captured of the phantom. The fused silica wafer was then replaced with the wafer onto which the 200  μm-thick adipose film was mounted and another FL image was captured. The film was then replaced with the next-thickest film and imaging was repeated. Thicker films were added and imaged until no more red FL was visible through the film’s thickness. Since films up to 1 mm were fabricated, if there was FL visible at depths greater than this distance, films were stacked to continue increasing the film thickness in 100  μm increments.

This imaging procedure was repeated using the connective tissue phantom base and connective thin films. Sample images collected during the data collection procedure are shown in Figs. 6(e) and 6(f) for a connective phantom with a 400  μm-thick film applied and an adipose phantom with an 800  μm-thick film applied, respectively.

#### 2.5.3 Image analysis

The image analysis aimed to determine the detection sensitivity of the Eagle device to each of the concentrations and depths.

Once all images were collected, the wells were classified as saturated (SAT), FL, or not fluorescent (NFL). Since the relative position of the phantom and the imaging device was constant for each of the adipose and connective imaging sets, a binary mask was created using the first image of each set using Matlab (MathWorks, Natick, Massachusetts, United States). Circular ROIs were used to outline each well to create the mask. Each well in each image was evaluated individually before holistic image assessment and trend observation across images.

The wells were classified using rules and thresholds in the xyY color space. First, the luminance of the well ($Y_{\text{well}}$) was compared to a luminance threshold ($Y_{\mathrm{SAT}}$) to determine whether or not the well was SAT. If not SAT, then ΔE was measured in reference to the control well of the same size and compared to a threshold; ΔE values above the threshold were classified as FL and the rest were NFL.

## 3 Discussion

The phantom model that was developed can be used to measure the imaging performance of an FL imaging device relative to the six expert-recommended device features.^11^ In particular, recommendation (iii)—high sensitivity to a fluorophore of interest—was emphasized to provide a phantom model that can elucidate the sensitivity of an imaging device to detect fluorophores in clinically relevant quantities and geometries. Fluorophore types and quantities, tissue depths, and tissue-mimicking materials can be customized for use with different imaging devices, excitation wavelengths, and clinical applications.

### 3.1 Strengths and Limitations of the Phantom Model

In Sec. 1.1, requirements for the TPMs, fluorophore chip, and thin films of the phantom were defined. Evaluating the conformance to these requirements revealed the strengths and limitations of the phantom model.

#### 3.1.1 Tissue phantom material

The phantom was designed with ease of fabrication in mind using low-cost methods and materials and without the necessity for specialized skills, equipment, or fabrication methods. The TPMs were also designed to be adaptable to a desired clinical application or tissue type. Iteration to select the ideal absorbers and scatterers in proportions that satisfy optical property and FL requirements may be time-consuming. However, the process can be accelerated by fabricating small volumes of phantoms in many iterations simultaneously (e.g., in a 96-well plate). Small volumes will set quickly, facilitating rapid iteration.

Limitations of TPMs were revealed during storage of the phantom. The phantom will dry out over time, causing it to harden and the absorption and scattering coefficients to increase. The phantoms should be stored at 4°C, shielded from light, and ideally in a form-fitting airtight container to slow the drying process. Storing the phantoms accordingly can prevent the phantoms from drying out for several months. Storage under various conditions and evaluation of long-term optical property stability was not evaluated, although this has been previously studied for hydrogel phantoms.^31^

#### 3.1.2 Fluorophore chip

The chip may be tedious to fabricate in the configuration presented but can be simplified. For example, if well size is not a variable of interest, the fluorophore chip can be fabricated using only the 3D printed mold, which creates the 5 mm-diameter wells, without the needles. This would also eliminate the capillary tubes, which take time to cleave and insert into the chip. Thin films may be eliminated if depth below the surface is not a variable of interest. For applications where a simple image of a fluorophore against a tissue-mimicking background is required (e.g., A/B testing or qualitative image quality assessments), this streamlined fluorophore chip can be fabricated and filled with ease.

#### 3.1.3 Future work

The phantom has some limitations that may be addressed in the future. First, the phantom does not currently include a method of replicating the optical properties of the tissues that the selected fluorophore would resemble. For example, for the phantom model to be used to measure the performance of an FL imaging device during breast cancer surgeries following administration of 5-ALA, it may be desirable for the fluorophore wells to mimic the optical properties (absorption and scattering) of breast carcinoma, in addition to mimicking the FL. Although the phantom as described does not explicitly achieve this, it may be possible to directly mix liquid absorbers and scatterers into the fluorophore loaded into the chip. Following this investigation, it may be desirable to vary the absorber and scatterer concentrations rather than the fluorophore concentration to examine how these affect the fluorophore’s FL. These data may reveal the ability of an imaging device to detect the fluorophore within tumors or tissues with varying optical properties to define the device’s imaging limitations more thoroughly.

The phantom may also be improved by adopting an alternative baffling method for eliminating light intrusion from the largest wells into neighboring wells. The existing method—using opaque black colorants—may cause absorption of light from the large wells, thus artificially decreasing the FL detected. Modifying the chip design so that the wells are surrounded with TPM rather than transparent PDMS would best replicate the clinical imaging scenario and eliminate the need for introducing opaque colorants. To accomplish this, a material, such as silicone, could be investigated for use as both the chip and TPM matrices. This would increase the strength and shelf-stability of the phantoms compared to gelatin while providing more realistic light-tissue interactions between the chip wells and surrounding tissue compared to PDMS.

### 3.2 Significance

The phantom was designed to measure the performance of an FL imaging device. To our knowledge, this is the only visible-wavelength phantom model capable of measuring imaging sensitivity with respect to depth below the surface, target size, and fluorophore concentration. This is also the only phantom model developed for visible light imaging devices with explicit customizability for other wavelengths. Consequently, this adaptable phantom model capable of assessing various imaging systems may influence development of a consistent standard of FL imaging device evaluation. The phantom can assess whether a particular FL imaging device can achieve the six previously listed expert-recommended requirements for an ideal FL imaging system.^11^ We have listed how the phantom may be used to evaluate an imaging device against these requirements.

1. Overlay WL and FL: the phantom can be used to test whether an imaging device’s overlay performs on different tissue backgrounds and to facilitate measurement of the minimum quantities (target size and concentration) that can be successfully detected and overlaid.
2. Operation under ambient room lighting conditions: a device’s ambient light imaging capability can be evaluated by imaging the phantom in ambient and dark room conditions and comparing the sensitivity in the two scenarios.
3. High sensitivity to fluorophore of interest: the phantom can be used to quantify the minimum detectable concentration and target size that an imaging device can detect at multiple depths below the surface.
4. Ability to quantify fluorophores: since the concentration of fluorophores in each phantom well is known, an imaging system’s quantification abilities may be verified by comparing the known versus measured concentrations, including below the surface and for different well sizes.
5. Ability to image multiple fluorophores simultaneously: the intrinsic fluorophores in the tissue phantom base and the fluorophore within the chip can be different. Alternatively, the chip can be loaded with multiple fluorophores, or additional fluorophores can be incorporated into the phantom material.
6. Maximized ergonomic use: the tissue phantom material can be molded into the desired anatomy (e.g., breast cavity) to evaluate ergonomics while imaging.

## Data, Materials, and Code Availability

Data are available upon request from the corresponding author (RSD).

## References

[1] Delpy D. T. et al., “Estimation of optical pathlength through tissue from direct time of flight measurement,” Phys. Med. Biol. 33(12), 1433–1442 (1988). PHMBA7 0031-9155 10.1088/0031-9155/33/12/008 3237772

[r2] Wilson B. C. Jacques S. L., “Optical reflectance and transmittance of tissues: principles and applications,” IEEE J. Quantum Electron. 26(12), 2186–2199 (1990). IEJQA7 0018-9197 10.1109/3.64355

[r3] Shupletsov E. et al., “Tissue mimicking phantoms for fluorescence imaging,” Proc. SPIE 11457, 41–46 (2020). PSISDG 0277-786X 10.1117/12.2564389

[r4] Madsen S. J. Patterson M. S. Wilson B. C., “The use of India ink as an optical absorber in tissue-simulating phantoms,” Phys. Med. Biol. 37(4), 985–993 (1992). PHMBA7 0031-9155 10.1088/0031-9155/37/4/012 1589459

[r5] Pleijhuis R. et al., “Tissue-simulating phantoms for assessing potential near-infrared fluorescence imaging applications in breast cancer surgery,” J. Visualized Exp. (91), 2–7 (2014). 10.3791/51776 PMC4828112 25286185

[r6] Ntombela L. Adeleye B. Chetty N., “Low-cost fabrication of optical tissue phantoms for use in biomedical imaging,” Heliyon 6, e03602 (2020). 10.1016/j.heliyon.2020.e03602 32258463 PMC7096755

[r7] Anastasopoulou M. et al., “Comprehensive phantom for interventional fluorescence molecular imaging,” J. Biomed. Opt. 21(9), 091309 (2016). JBOPFO 1083-3668 10.1117/1.JBO.21.9.091309 27304578

[r8] Patterson M. S. Chance B. Wilson B. C., “Time resolved reflectance and transmittance for the non-invasive measurement of tissue optical properties,” Appl. Opt. 28, 2331 (1989). APOPAI 0003-6935 10.1364/AO.28.002331 20555520

[9] Firbank M. Delpy D. T., “A design for a stable and reproducible phantom for use in near infra-red imaging and spectroscopy,” Phys. Med. Biol. 38(6), 847–853 (1993). PHMBA7 0031-9155 10.1088/0031-9155/38/6/015 7652018

[10] Hernandez-Quintanar L. Rodriguez-Salvador M., “Discovering new 3D bioprinting applications: analyzing the case of optical tissue phantoms,” Int. J. Bioprint. 5(1), 178 (2019). 10.18063/IJB.v5i1.178 32596533 PMC7294689

[11] DSouza A. V. et al., “Review of fluorescence guided surgery systems: identification of key performance capabilities beyond indocyanine green imaging,” J. Biomed. Opt. 21(8), 080901 (2016). JBOPFO 1083-3668 10.1117/1.JBO.21.8.080901 27533438 PMC4985715

[12] Ottolino-Perry K. et al., “Intraoperative fluorescence imaging with aminolevulinic acid detects grossly occult breast cancer: a phase II randomized controlled trial,” Breast Cancer Res. 23, 1–20 (2021). BCTRD6 10.1186/s13058-021-01442-7 34253233 PMC8276412

[13] Wang Q. et al., “Measurement of internal tissue optical properties at ultraviolet and visible wavelengths: Development and implementation of a fiberoptic-based system,” Opt. Express 16, 8685 (2008). OPEXFF 1094-4087 10.1364/OE.16.008685 18545582

[14] Sekar S. K. V. et al., “Diffuse optical characterization of collagen absorption from 500 to 1700 nm,” J. Biomed. Opt. 22(1), 015006 (2017). JBOPFO 1083-3668 10.1117/1.JBO.22.1.015006 28138693

[15] Bashkatov A. N. et al., “Optical properties of the subcutaneous adipose tissue in the spectral range 400-2500 nm,” Opt. Spectrosc. 99(5), 836–842 (2005). OPSUA3 0030-400X 10.1134/1.2135863

[16] Prahl S. A. van Gemert M. J. C. Welch A. J., “Determining the optical properties of turbid media by using the adding–doubling method,” Appl. Opt. 32(4), 559 (1993). APOPAI 0003-6935 10.1364/AO.32.000559 20802725

[17] Taroni P. et al., “Breast tissue composition and its dependence on demographic risk factors for breast cancer: non-invasive assessment by time domain diffuse optical spectroscopy,” PLoS ONE 10, e0128941 (2015). POLNCL 1932-6203 10.1371/journal.pone.0128941 26029912 PMC4452361

[18] Jacques S. L., “Optical properties of biological tissues: a review,” Phys. Med. Biol. 58(14), 5007–5008 (2013). PHMBA7 0031-9155 10.1088/0031-9155/58/14/5007 23666068

[19] Peters V. G. et al., “Optical properties of normal and diseased human breast tissues in the visible and near infrared,” Phys. Med. Biol. 35(9), 1317–1334 (1990). PHMBA7 0031-9155 10.1088/0031-9155/35/9/010 2236211

[20] Berns R. S. et al., “Visual determination of suprathreshold color-difference tolerances using probit analysis,” Color Res. Appl. 16, 297–316 (1991). CREADU 0361-2317 10.1002/col.5080160505

[21] Luo M. R. Cui G. Rigg B., “The development of the CIE 2000 colour-difference formula: CIEDE2000,” Color Res. Appl. 26, 340–350 (2001). CREADU 0361-2317 10.1002/col.1049

[22] Sharma G. Wu W. Dalal E. N., “The CIEDE2000 color-difference formula: implementation notes, supplementary test data, and mathematical observations,” Color Res. Appl. 30, 21–30 (2005). CREADU 0361-2317 10.1002/col.20070

[23] Vardaki M. Z. et al., “Studying the distribution of deep Raman spectroscopy signals using liquid tissue phantoms with varying optical properties,” Analyst 140, 5112–5119 (2015). ANLYAG 0365-4885 10.1039/C5AN01118C 26075989

[24] De Grand A. M. et al., “Tissue-like phantoms for near-infrared fluorescence imaging system assessment and the training of surgeons,” J. Biomed. Opt. 11(1), 014007 (2008). 10.1117/1.2170579 PMC2486408 16526884

[25] Michels R. Foschum F. Kienle A., “Optical properties of fat emulsions,” Opt. Express 16, 5907 (2008). OPEXFF 1094-4087 10.1364/OE.16.005907 18542702

[26] Prahl S. A., “Tabulated molar extinction coefficient for hemoglobin in water,” https://omlc.org/spectra/hemoglobin/summary.html (1998).

[27] Walger E. et al., “Study of the direct red 81 Dye/Copper(II)-phenanthroline system,” Mol. A J. Synth. Chem. Nat. Prod. Chem. 23(2), 242 (2018). 10.3390/molecules23020242 PMC6017580 29370132

[28] Kim A. et al., “Quantification of in vivo fluorescence decoupled from the effects of tissue optical properties using fiber-optic spectroscopy measurements,” J. Biomed. Opt. 15(6), 067006 (2010). JBOPFO 1083-3668 10.1117/1.3523616 21198210 PMC3025598

[29] Piruska A. et al., “The autofluorescence of plastic materials and chips measured under laser irradiation,” Lab Chip 5(12), 1348–1354 (2005). LCAHAM 1473-0197 10.1039/b508288a 16286964

[30] Cook J. R. Bouchard R. R. Emelianov S. Y., “Tissue-mimicking phantoms for photoacoustic and ultrasonic imaging,” Biomed. Opt. Express 2(11), 3193 (2011). BOEICL 2156-7085 10.1364/BOE.2.003193 22076278 PMC3207386

[31] Hacker L. et al., “Criteria for the design of tissue-mimicking phantoms for the standardization of biophotonic instrumentation,” Nat. Biomed. Eng. 6(5), 541–558 (2022). 10.1038/s41551-022-00890-6 35624150

